# Role of prostate cancer stem-like cells in the development of antiandrogen resistance

**DOI:** 10.20517/cdr.2022.07

**Published:** 2022-06-01

**Authors:** Prem Prakash Kushwaha, Shiv Verma, Shashank Kumar, Sanjay Gupta

**Affiliations:** ^1^Department of Urology, Case Western Reserve University, Cleveland, OH 44106, USA.; ^2^The Urology Institute, University Hospitals Cleveland Medical Center, Cleveland, OH 44106, USA.; ^3^Molecular Signaling and Drug Discovery Laboratory, Department of Biochemistry, Central University of Punjab, Bathinda 151401, India.; ^4^Department of Pathology, Case Western Reserve University, Cleveland, OH 44106, USA.; ^5^Department of Pharmacology, Case Western Reserve University, Cleveland, OH 44106, USA.; ^6^Department of Nutrition, Case Western Reserve University, Cleveland, OH 44106, USA.; ^7^Divison of General Medical Sciences, Case Comprehensive Cancer Center, Cleveland, OH 44106, USA.

**Keywords:** Prostate cancer, second-generation antiandrogens, cancer stem cells, castration resistance prostate cancer, androgen deprivation therapy

## Abstract

Androgen deprivation therapy (ADT) is the standard of care treatment for advance stage prostate cancer. Treatment with ADT develops resistance in multiple ways leading to the development of castration-resistant prostate cancer (CRPC). Present research establishes that prostate cancer stem-like cells (CSCs) play a central role in the development of treatment resistance followed by disease progression. Prostate CSCs are capable of self-renewal, differentiation, and regenerating tumor heterogeneity. The stemness properties in prostate CSCs arise due to various factors such as androgen receptor mutation and variants, epigenetic and genetic modifications leading to alteration in the tumor microenvironment, changes in ATP-binding cassette (ABC) transporters, and adaptations in molecular signaling pathways. ADT reprograms prostate tumor cellular machinery leading to the expression of various stem cell markers such as Aldehyde Dehydrogenase 1 Family Member A1 (ALDH1A1), Prominin 1 (PROM1/CD133), Indian blood group (CD44), SRY-Box Transcription Factor 2 (Sox2), POU Class 5 Homeobox 1(POU5F1/Oct4), Nanog and ABC transporters. These markers indicate enhanced self-renewal and stemness stimulating CRPC evolution, metastatic colonization, and resistance to antiandrogens. In this review, we discuss the role of ADT in prostate CSCs differentiation and acquisition of CRPC, their isolation, identification and characterization, as well as the factors and pathways contributing to CSCs expansion and therapeutic opportunities.

## INTRODUCTION 

Prostate cancer is the most commonly diagnosed cancer in the United States, and approximately 50% of the men diagnosed with advance stage prostate cancer undergo androgen deprivation therapy (ADT)^[1]^. US Food and Drug Administration approved ADT to treat metastatic prostate cancer as a neo-adjuvant in post-radiation therapy^[2]^. ADT has also been accepted as the first-line treatment for prostate tumors that have extended to the lymph nodes, and biochemical recurrence followed by prostate-specific antigen (PSA) resurgence, asymptomatic metastatic and locally advanced disease^[3]^. Since androgen receptor (AR) is essential for the function, survival, and differentiation of prostatic tissue, ADT reduces androgens necessary to block cancer progression^[4]^. Accumulating data suggests that androgens are important players in the human body in maintaining physiological functions^[5-6]^. Androgen receptor holds a key function in prostatic epithelial cells’ growth and proliferation in response to testosterone^[7]^. ADT comprises the use of the first-generation antiandrogens such as bicalutamide, nilutamide or flutamide that solely targets AR translocation to the nucleus and prevent downstream signaling. The second-generation antiandrogens *viz*. enzalutamide, apalutamide and darolutamide, as well as inhibitors of androgen biosynthesis such as abiraterone acetate, improve upon this mechanism^[8]^. Changes in the function of AR signaling result in tumor suppression to tumor promotion, where the disease eventually progresses to the emergence of castration-resistant prostate cancer (CRPC)^[9]^. Accumulated evidence suggests that ADT has a significant role in the management of metastatic prostate cancer; it reduces complications and enhances overall survival^[2,10-11]^. In a study based on Cochrane meta-analysis, the neo-adjuvant ADT with radical prostatectomy significantly improves adverse histopathologic parameters such as surgical margin or pathologic tumor stage^[12]^. Studies reported that prostate cancer patients undergoing radiotherapy together with ADT treatment further increases the probability of disease-free survival^[12-13]^. This combinatorial treatment is well accepted and highlighted in the guidelines of the American Urological Association and European Association of Urology^[14-15]^. However, numerous studies indicate that ADT is associated with a multitude of side effects that can impact the quality of life^[16-18]^. These include fatigue, loss of libido, arterial stiffness, erectile dysfunction, hot flushes, new-onset diabetes mellitus, altered body composition, osteoporosis and induced skeletal complications, and cognitive decline^[16-18]^. Some recent findings also demonstrated that ADT treatment might increase cardiovascular-mediated morbidity and mortality^[19-20]^. 

Prostate cancer resistance during ADT treatment is reported in *in vitro* models of recurrence and CRPC patients^[21]^. CRPC development is linked with genes associated with AR signaling, both at transcription and translation levels^[21]^. A study including multiple isogenic tumor xenograft models demonstrated increased AR expression in recurrent tumor samples compared to paired androgen-sensitive samples^[22]^. AR stabilization alters the rate of post-translational modifications and interaction with heat shock proteins which ultimately modulates normal cellular physiology^[23]^. Studies also reported that stabilization of AR is positively associated with ADT resistance, which may be linked with CRPC^[4,24]^. Phosphorylation at particular sites and enrichment of growth factors reactivate AR, which further increases prostate cancer proliferation under low androgen levels^[25]^. Apart from AR stabilization and phosphorylation, the mutation in *AR* gene is also associated with resistance development^[26]^. A point mutation was reported in the lymph node of a patient with metastatic prostate cancer and causes amino acid substitution at position 878, threonine to alanine^[27]^. This amino acid substitution results in response to non-androgen hormones that enhance resistance to ADT treatment. Some other reported mutations are W742C, H875Y/T, F877L and L702H in response to resistance development against enzalutamide, nilutamide, flutamide and bicalutamide^[28-29]^. Other factors such as different AR splice variant expression, aberrant glucocorticoids and glucocorticoid receptor expression, impairment of DNA repair pathway, miRNAs, cellular metabolism, and alterations in enzymatic and signaling pathways tightly support resistance development in response to ADT^[30-31]^.

Emerging research implicates that cancer stem cells (CSCs) are key to the development of therapeutic resistance, and studies have also established a link to ADT relapse tumors^[32]^. Subsequent work has shown that ADT increases the enrichment of CSCs populations which are inherently treatment-resistant having the ability to promote CRPC^[33]^. The CSC hypothesis is an emerging model that describes several molecular characteristics of cancer. CSCs facilitate the development of a cellular hierarchy, maintain a CSC rich-pool, differentiate into a proliferating progeny, and assist the formation of a heterogeneous tumor^[34]^. As a result, there is a strong consensus that CSCs are the cells of origin in cancer and have the propensity of cancers to relapse, metastasize, and develop resistance to conventional therapies^[34]^. In this review, we describe the role of ADT in CSC differentiation during CRPC acquisition, their isolation, identification and characterization. Outlining the underlying mechanisms triggered by ADT and highlighting potential CSCs targets could aid in the development of future therapeutic strategies in prostate cancer patients leading to improved outcomes.

## PROSTATE CANCER STEM-LIKE CELLS

### Stem cell markers in prostate cancer 

Normal stem cells possess the intrinsic capacity for unlimited replicative potential as well as differentiation into all lineages of mature cells required for tissue and/or organ maintenance^[35]^. In general, cancer cells possess cellular heterogeneity and inherent genetic instability, which makes them immortal in characteristics. CSCs are commonly defined as cells that demonstrate characteristics similar to a normal stem cell, including lack of senescence, self-renewal capacity and pluripotency^[36]^. This type of cancer cell possesses the ability to develop a cellular hierarchy, facilitating the maintenance of a CSC pool while differentiating into a proliferating progeny, enabling the formation and growth of a heterogeneous tumor.

The prostate gland consists of basal (undifferentiated), differentiated and neuroendocrine cells^[37]^. The basal cells are androgen-independent and express cytokeratin 5, cytokeratin 14, Tumor Protein P63, and Cell Surface Glycoprotein (CD44) markers^[38-40]^. They also express much less AR, PSA and prostatic acid phosphatase (PAP)^[38-40]^. Differentiated cells include glandular epithelial and secretory luminal cells, which express AR, PSA, PAP, cytokeratin 8 and cytokeratin 18^[41]^. The neuroendocrine cells are androgen-independent and do not secrete PSA^[42]^. Prostate cancer stem cells are androgen-independent cells that give rise to androgen-sensitive progenitor cells^[38]^. These progenitor cells in the presence of androgens differentiate into androgen-dependent cells. A number of research studies indicate that CSCs are considered as the cells of origin in cancer and have been linked to tumorigenesis, treatment resistance and cancer relapse^[41]^.

Several research studies identified prostate CSCs genes that are important for self-renewal, pluripotency, resistance and serve as markers for identification^[43]^. Stem cell antigen-1, aldehyde dehydrogenases (ALDH), CD133 (PROM1), trophoblast cell surface antigen 2 and CD44 are markers to identify prostate CSCs in the basal compartment^[44]^. The other common markers include CD44, CD24, and CD49 Antigen-Like Family Member D (also known as Integrin Subunit Alpha 4), which have been tightly associated with prostate CSCs^[45]^. Another study identified a rare prostate cancer stem cell maker, KIT Proto-Oncogene, Receptor Tyrosine Kinase (KIT) in adult mouse prostatic stem cell population, which possess cancer stem cell-like features such as differentiation and self-renewal^[46]^. The prostate CSCs enriched in CD133+ cells isolated from established primary human prostate cancer cell lines and in alpha(2)beta(1)-integrin subunit phenotype identified as genetically unstable with clonogenic formation and invasive potential^[47]^. Yu *et al*.^[48]^ have identified high ALDH1 activity in LNCaP and PC-3 prostate cancer cells associated with CSC-like properties; in particular, ALDH^hi^/CD44^+^ cells possess a high clonogenic function and tumorigenic potential. A study conducted on prostate cancer tissue specimens indicated *Oct4*, *Sox2* and *Nanog* genes as prostate CSC markers^[49]^. Collins *et al*.^[50]^ further demonstrate in the mouse xenograft model that elevated levels of ALDH support stemness in cells; indeed, ALDH^hi^/CD44^+^/α2β1^+^ cells are enhanced during castration and were critical in the development of antiandrogen resistance. Further, cells possessing similar phenotypes were isolated from clinical specimens and analyzed for self-renewal and spheroid formation. The outcome showed that ALDH^hi^/CD44^+^/α2β1^+^ cells significantly support cell proliferation and colony formation^[51]^. A study performed on patient biopsies samples (Gleason score range 5-6) suggested that CD133^+^/CD44^+^/ATP-binding cassette sub-family G member 2 (ABCG2)^+^/CD24^− ^cells actively participate in spheroid formation^[52]^. In addition, primary tumor cells containing Enhancer of Zeste 2 Polycomb Repressive Complex 2 Subunit (EZH2^+^)/E-cadherin^-^ markers are highly associated with tumor recurrence [Table 1]^[53]^.

**Table 1:** Prostate cancer stem cells markers

**Pathways**	**Markers names**	**Gene symbol**	**Ref.**
Tumor progression	KIT proto-oncogene, receptor tyrosine kinase	*CD117/c-kit*	[43,54-56]
	Prominin 1	*CD133*	
	Indian blood group	*CD44*	
	α2β1 integrin	*ITGB1*	
	Integrin Subunit Alpha 6	*α6 integrin*	
	C-X-C motif chemokine receptor 4	*CXCR4*	
	Epithelial cell adhesion molecule	*EPCAM*	
	Cytokeratin 5	*KRT5*	
	Kallikrein related peptidase 3	*KLK3/PSA*	
	Tumor-associated calcium signal transducer 2	*Trop2*	
	Activated leukocyte cell adhesion molecule	*ALCAM*	
	Aldehyde dehydrogenase 1 family member A1	*ALDH1*	
	Transglutaminase 2	*TG2*	
	Enhancer of zeste 2 polycomb repressive complex 2 subunit	*EZH2*	
Metastatic colonization and growth	KIT proto-oncogene, receptor tyrosine kinase	*CD117/c*	[43,56]
	C-X-C motif chemokine receptor	*CXCR4*	
	Epithelial cell adhesion molecule	*EPCAM*	
	E-cadherin/ cadherin 1	*CDH1*	
	Indian blood group	*CD44*	
	Enhancer of zeste 2 polycomb repressive complex 2 subunit	*EZH2*	
Recurrence and therapeutic resistance	KIT proto-oncogene, receptor tyrosine kinase	*CD117/c-kit*	[43,54-56]
	α2β1 integrin	*ITGB1*	
	Integrin subunit alpha 6	*ITGA6*	
	E-cadherin/ cadherin 1	*CDH1*	
	Epithelial cell adhesion molecule	*EPCAM*	
	C-X-C motif chemokine receptor 4	*CXCR4*	
	Enhancer of zeste 2 polycomb repressive complex 2 subunit	*EZH2*	
	Aldehyde dehydrogenase 1 family member A1	*ALDH1*	
	Transglutaminase 2	*TG2*	
	Activated leukocyte cell adhesion molecule	*CD166/ALCAM*	
	Kallikrein related peptidase 3	*KLK3/PSA*	
	Androgen receptor splice variant 7	*AR-V7*	
	ATP binding cassette subfamily G member 2 (junior blood group)	*ABCG2*	
Self-renewal capacity	Prominin 1	*CD133*	[43,55]
	Cytokeratin 5	*KRT5*	
	Kallikrein related peptidase 3	*KLK3/PSA*	
	Aldehyde dehydrogenase 1 family member A1	*ALDH1*	
	Activated leukocyte cell adhesion molecule	*CD166/ALCAM*	
	C-X-C motif chemokine receptor 4	*CXCR4*	
	Tumor-associated calcium signal transducer 2	*Trop2*	
	Integrin subunit alpha 6	*α6 integrin*	
	α2β1 integrin	*ITGB1*	
	Indian blood group	*CD44*	
Stemness gene expression	Prominin 1	*CD133*	[43,54]
	Indian blood group	*CD44*	
	E-cadherin/ cadherin 1	*CDH1*	
	Kallikrein related peptidase 3	*KLK3/PSA*	
	Aldehyde dehydrogenase 1 family member A1	*ALDH1*	
	Enhancer of zeste 2 polycomb repressive complex 2 subunit	*EZH2*	

### Molecular pathways in the generation of prostate cancer stem-like cells post ADT

**Figure 1:**
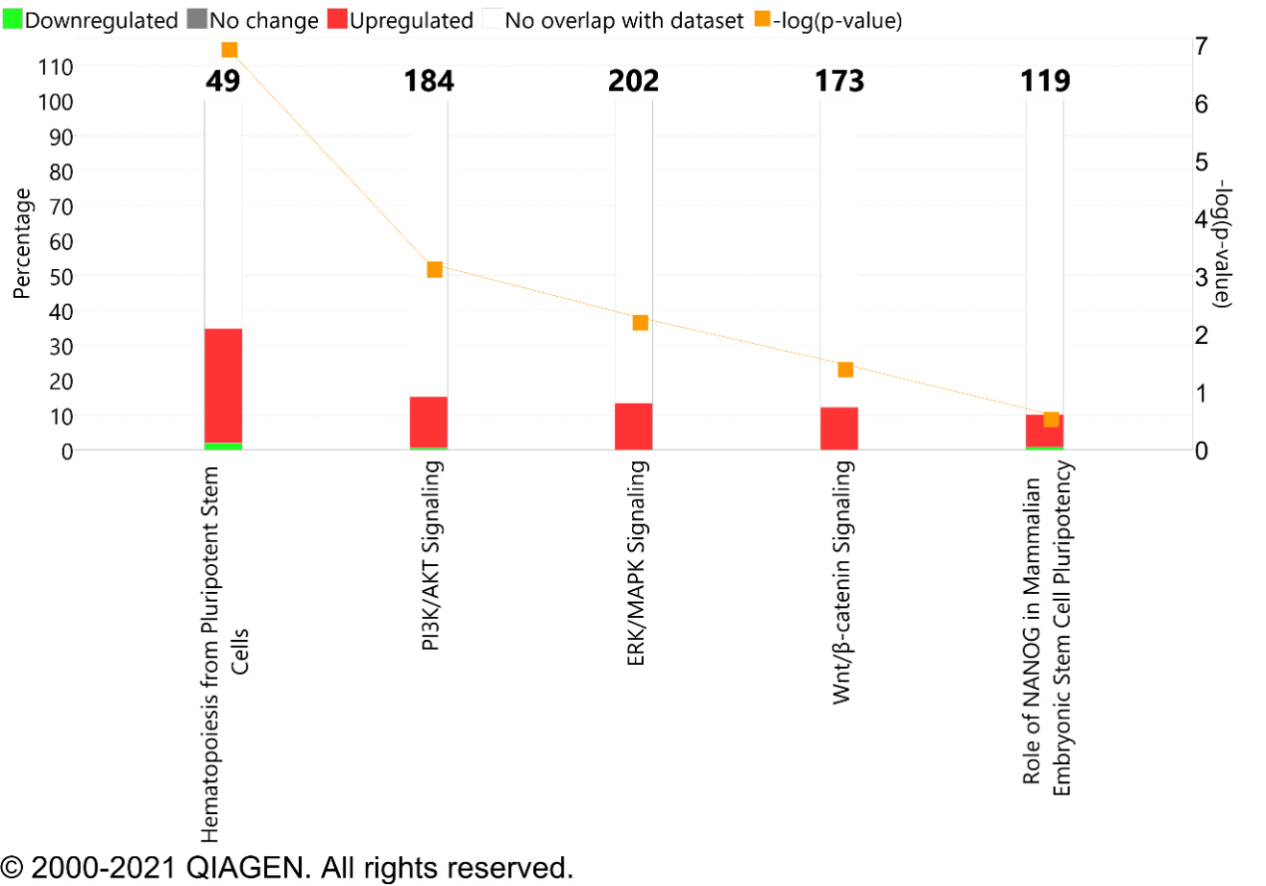
Canonical signaling pathway overrepresented in prostate cancer patients under ADT. These pathways play a critical role in driving cancer stem-like cell phenotype. The red bar represents the genes upregulated and green is downregulated and overlaid with IPA database. The X-axis in the graph represents the signaling pathways while the Y-axis showed -log (*P*-value) and percentage (%). ADT: Androgen deprivation therapy

AR is a key transcription factor involved in androgen-dependent prostate cancer growth. Targeting AR with the first-generation antiandrogens does not inhibit inter or intramolecular N-C interactions required for the nuclear localization^[57]^. At the diagnosis of metastatic CRPC, the common genomic alteration event found in AR is amplification and AR mutation^[58]^. These AR genomic alterations dysregulate the signaling pathway in patients and demonstrate a compensatory resistance mechanism *via* increasing AR expression in response to the potent AR inhibition by enzalutamide, which results in diminished efficacy of treatment overtime^[59]^. In a systemic study, exome sequencing of 150 metastatic CRPC biopsy specimens demonstrated 63% of AR mutation and amplification in comparison to 440 primary prostate cancer tissues^[60]^. Apart from AR mutation, AR variants such as ARV7 were also reported for resistance development and support androgen-independent growth of prostate cancer cells^[61]^. Prostate cancer patients who underwent ADT showed hematopoiesis from pluripotent stem cells, PI3K/AKT signaling, ERK/MAPK signaling, and Wnt/β-catenin signaling, and the role of Nanog in mammalian embryonic stem cell pluripotency signaling pathways were overrepresented [Figure 1]. This information revealed that the genomic alteration in AR either by amplification or mutation tends to increase the expression of associated stem cell markers.

With reference to hematopoiesis, pluripotent stem cells, the expression of transmembrane receptors genes, which includes *CD4*, *CD247*, *CD3E*, *CD8A*, *CSF3*, *CXCL8*, and family members of immunoglobulin heavy constant gamma proteins (IGHA1, IGHD, IGHG1, IGHG2, IGHG3, IGHM) and the expression of cytokines such as IL6 and IL10 were increased in patients undergoing ADT [Table 2]. 

**Table 2:** List of genes, location, types, along with fold changes value associated with hematopoiesis pluripotent stem cells

**Gene symbol**	**Gene name**	**Fold change in ADT **	**Location**	**Type(s)**
*CD4*	CD4 molecule	1.829	Plasma membrane	transmembrane receptor
*CD247*	CD247 molecule	1.437	Plasma membrane	transmembrane receptor
*CD3E*	CD3e molecule	1.597	Plasma membrane	transmembrane receptor
*CD8A*	CD8a molecule	1.469	Plasma membrane	other
*CSF3*	colony stimulating factor 3	5.074	Extracellular space	cytokine
*CXCL8*	C-X-C motif chemokine ligand 8	3.395	Extracellular space	cytokine
*FCER1G*	Fc fragment of IgE receptor Ig	1.815	Plasma membrane	transmembrane receptor
*IGHA1*	immunoglobulin heavy constant alpha 1	3.2	Extracellular space	other
*IGHD*	immunoglobulin heavy constant delta	2.58	Extracellular space	other
*IGHG1*	immunoglobulin heavy constant gamma 1 (G1m marker)	2.139	Extracellular space	other
*IGHG2*	immunoglobulin heavy constant gamma 2 (G2m marker)	3.362	Plasma membrane	other
*IGHG3*	immunoglobulin heavy constant gamma 3 (G3m marker)	2.922	Extracellular space	other
*IGHM*	immunoglobulin heavy constant mu	2.946	Plasma membrane	transmembrane receptor
*IL6*	interleukin 6	6.333	Extracellular space	cytokine
*IL10*	interleukin 10	1.764	Extracellular space	cytokine
*LIF*	LIF interleukin 6 family cytokine	2.636	Extracellular space	cytokine

### Isolation, identification and characterization of prostate cancer stem cells

Extensive work has shown that CSCs from primary prostate tumors or established cancer cell lines can be isolated from a heterogeneous population using cell surface markers, such as CD44 and CD133, aldehyde dehydrogenase (ALDH) activity using the ALDEFLUOR assay, and Hoechst dye to identify the side population^[62-63]^. Isolation of prostate CSCs has been performed by several groups. The first CSC isolation was performed from patients undergoing radical prostatectomy^[64]^. These CSCs exhibited a significant capacity for self-renewal and the ability to regenerate the phenotypically mixed populations of non-clonogenic cells. The prostate CSCs were isolated using the CD44/α2β1^high^/CD133^+^ phenotype and demonstrated high clonogenic and invasive capacity of basal cell origin with high levels of genetic instability^[64-65]^. A study by Rajasekhar *et al*.^[66]^ observed that CSCs expressing human pluripotent stem cell marker TRA-60-1^+^ /CD151^+^ /CD166^+^ possess high self-renewal and differentiation ability, and were able to reiterate tumor heterogeneity in serial *in vivo* transplantations. More recent studies have identified that a majority of prostate tumors harbor prostate-specific *TMPRSS2* gene and the *ERG* oncogene (*TMPRSS2: ERG*) gene fusion which could be used as a stem cell marker with high specificity providing ERG-driven survival advantages^[67]^. Moreover, ALDH1^high^ prostate cancer cells have been shown to exhibit several CSC characteristics such as clonogenicity, migration, tumorigenicity, and propensity to form metastases *in vivo*^[68]^. Another method to enrich the CSC population is developing tumorspheres in cell culture with a higher *in vivo* tumor incidence rate^[69]^.

Our group and others have demonstrated fluorescence-activated cell sorting and magnetic-activated cell sorting utilizing various human prostate cancer cell lines^[70-71]^. Other studies showed that CD44 and CD133 were associated with high Nanog expression in prostate carcinoma cell lines^[71]^. Nanog has shown to be predominantly expressed from the *NanogP8 *pseudogene in a panel of prostate carcinoma cells including DU145, LNCaP, and PC-3 and primary prostate carcinoma cells. NanogP8 expression was enriched several folds in CD133^+^ and CD133^+^/CD44^+^ CSCs compared to non-CSCs^[71]^. Human prostate cancer PC-3 cells displayed high CD44^+^/CD133^+^ CSC-like features including enhanced tumor sphere formation and elevated Nanog levels. Similarly, CD117^+^/ABCG2^+^ cells isolated from 22Rv1 prostate cancer cells overexpress the core stem cell transcription factors, Nanog, Oct3/4, and Sox2, and the CSC marker CD133^[72]^. A recent study from our group has demonstrated high expression of ALDH1^high^, Oct4 and Sox2 in clinical prostate cancer specimens undergoing ADT, compared to grade-matched controls^[73]^.

Most standard therapies for prostate cancer primarily affect cancer cells, but CSCs undergo G0/G1 phase cell cycle arrest and remain static, thus evading cell death from chemotherapeutic drugs^[74]^. Experimental data also suggest that CSCs are resistant to conventional chemotherapy and radiation and may be the cells responsible for disease recurrence and/or progression^[75]^. A study showed that CD133^+ ^had a high capacity to proliferate *in vitro *and have AR^+ ^phenotype^[76]^. These CD133^+^ cells form branched spheroids structure in a 3D culture system and generate prostatic-like acini *in vivo*^[76]^. Hence, the drug-resistant characteristic of CSCs is useful to isolate and identify CSCs. Previous studies have shown that radiotherapy combined with hypoxic culture can also be used to enrich CSCs population^[77]^.

### Therapeutic opportunities for prostate cancer stem-like cells

**Figure 2:**
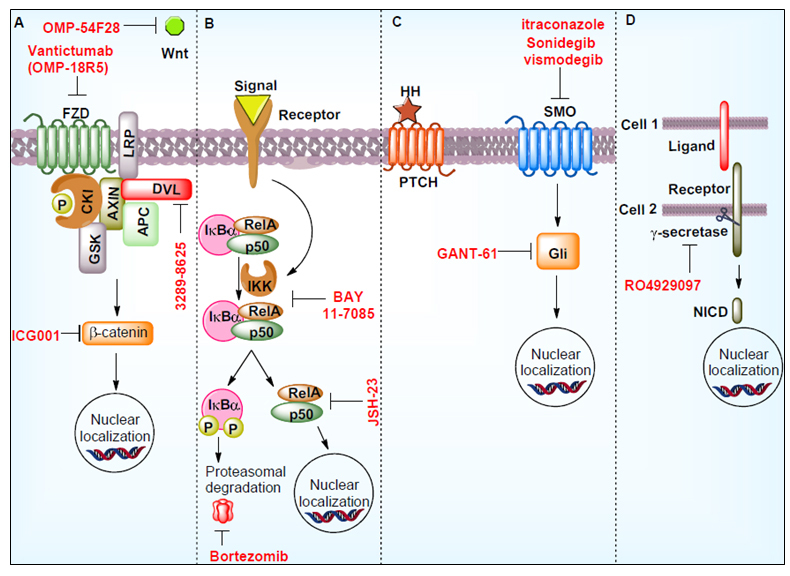
Signaling pathways regulating CSCs and the use of inhibitors in suppressing these pathways. These molecules thereof could be developed as potential therapeutics. The denotes in the figure are: Hedgehog signaling pathway (HH), notch intracellular domain (NICD), phosphorylation (P), smoothened (SMO), Wnt signaling pathways (Wnt), nuclear factor of kappa light polypeptide gene enhancer in B-cells inhibitor, alpha (IkBα), RELA Proto-Oncogene (RelA), Frizzled (FZD), casein kinase I (CKI), Axin (AXIN), APC Regulator of WNT Signaling Pathway (APC), Dishevelled (Dvl), Glycogen Synthase Kinase (GSK), IkappaB Kinase (IKK).

Prostate cancer patients undergo treatment therapy such as radiotherapy or chemotherapy, resulting in shrinkage of tumors^[78]^. However, after therapy, some cells accumulate genetic/epigenetic changes that result in loss of control on self-renewal potential. These cells, referred to as prostate CSCs, reprogram the tumor environment to their benefit, supporting increased survival, self-renewal, and tumor recurrence^[64]^. Research showed that cellular immunotherapy has some beneficial role in the treatment of prostate cancer^[79]^. The T cell-based immunotherapy showed a positive response to prostate cancer patients with metastatic CRPC and increased the overall survival^[79]^. A research group prepared an immunogenic peptide derived from dendritic cells sensitized to CD44 and EpCAM followed by co-culture with the expanded peripheral blood lymphocyte (PBL)-derived cytokine-induced killer cell^[80]^. This study showed that dendritic cells- cytokine-induced killer cells exhibit remarkable cytotoxicity against prostate cancer stem-like cells-enriched prostate spheroids both *in vitro* and *in vivo*^[80]^. In addition to these findings, several other cellular events impart growth advantages to CSCs. In this context, various signaling pathways such as Wnt/β-catenin, hedgehog, NF-κB and Notch; ABC transporters and tumor microenvironment could be the putative target(s) for prostate CSCs^[81] ^[Figure 2].

Wnt signaling pathway is involved in various cellular processes and is crucial for cell fate determination, cell polarity, cell migration, neural patterning and organogenesis during embryonic development. Wnt pathway is also associated with the maintenance of stem cells in a self-renewing state^[82]^. A study demonstrated that Wnt signaling activation is oncogenic in the prostate and supports CRPC growth *in vivo*^[83]^. This study also showed that increased Wnt signaling induces neuroendocrine differentiation, epithelial-mesenchymal transition and drives stem cell-like features to prostate cancer cells. A number of small molecule inhibitors and monoclonal antibodies have been tested to inhibit the Wnt signaling pathway. Wnt signaling inhibitors such as 3289-8625, Foxy-5, and OMP-54F28 have been reported to inhibit prostate cancer cell growth^[84-86]^. A porcupine (palmitoylation of Wnt ligands) inhibitor LGK974 combined with docetaxel and paclitaxel also showed remarkable effectiveness on solid tumors^[64]^. A monoclonal antibody vantictumab (OMP-18R5) blocks canonical Wnt signaling pathways and inhibits prostate cancer progression^[87]^.

Hedgehog signaling pathways play an important role in the development of prostate cancer. Hedgehog signaling targets genes involved in prostate CSCs survival, proliferation, and metastasis^[88]^. This signaling also enhanced the overexpression of ABC transporters in prostate cancer cells^[88]^. Hedgehog signaling inhibitor sonidegib (LDE-225) suppresses the key genes including *Oct4*, *Nanog*, *c-Myc*, and *Sox2* involved in self-renewal and stemness potential^[89]^. Gli transcription factor inhibitor GANT-61 inhibits PTCH1 expression and tumor growth *in vivo*^[90]^. Other Hedgehog signaling inhibitors such as vismodegib, itraconazole and orteronel either alone or in combination and/or surgery inhibit prostate cancer growth^[64]^.

Upregulation of the NF-κB pathway has been observed in cancer stem cells^[91]^. Various studies also demonstrated that NF-κB signaling was upregulated in prostate cancer cells and associated with increased progression, chemotherapy resistance, metastasis and recurrence^[92]^. Several clinical trials have been performed by targeting the NF-κB signaling pathway. Clinical trial NCT01695473 used PI3K inhibitor BKM120, which acts downstream of NF-κB in high-risk localized prostate cancer patients^[93]^. NCT00118092 clinical trial was performed using heat shock protein 90 inhibitor 17-(allylamino)-17-demethoxygeldanamycin to treat metastatic prostate cancer patients^[94]^. Aspirin, a reported drug for inflammation-regulated cancer, was utilized in clinical trial NCT02757365^[95]^. This trial demonstrated that aspirin suppresses CRPC progression.

Notch signaling pathways are well known for their contribution to self-renewal, differentiation, resistance and stemness development^[96]^. Interaction of Notch receptor and ligand facilitates NICD production through gamma-secretase, which translocates to the nucleus and initiates transcription of self-renewal, stemness, and other CSC development associated genes^[97]^. In this context, a number of gamma-secretase inhibitory agents were identified. A clinical trial (NCT01200810) was performed in prostate cancer patients using gamma-secretase inhibitor RO4929097 with bicalutamide^[98]^. This trial compares PSA expression with time after surgery/radiation and combined treatment^[98]^. 

Alteration in ABC transporter genes and tumor microenvironment tightly regulates cellular physiology and transcriptomic machinery in prostate CSCs^[99]^. The tumor microenvironment plays a decisive role in regulating CSCs progression^[100]^. It also facilitates abnormal cancer signaling pathways, epithelial-mesenchymal transition, invasion, *etc*. Overexpression of ABC transporters exports therapeutic drugs outside the cells, which makes them resistant to the drug^[101]^. Several lines of research have been performed to target the ABC transporters. An ABC transporter efflux inhibitor verapamil inhibits prostate cancer proliferation by inhibiting the potassium ion channel^[102]^. Cyclosporin A, another ABC transporter inhibitor, inactivates NFATc1 (nuclear factor of activated T-cells) in biochemical recurrence and CRPC^[103]^.

## CONCLUSION

Despite thorough research for mechanisms leading to CRPC as a result of resistance to antiandrogens in the past few decades, our understanding remains limited. ADT causes complex alterations within tumors in terms of factors and pathways as well as its epigenetics and genetics affecting the tumor microenvironment. Several research studies showed that aberrant cellular signaling, generation of AR variants, and AR mutation support the development of drug resistance. The accumulative effects of these factors also contribute to the generation of prostate cancer stem-like cells. CSCs are the reservoir of cancer cells that exhibit surface markers such as ALDH, CD133 and CD44, possessing properties of self-renewal and the ability to reestablish the heterogeneous tumor cell population promoting metastatic colonization, self-renewal, and recurrence. Research showed that targeting prostate CSCs could be a better strategy for the treatment of CRPC. In this direction, various small molecule inhibitors, antibodies and other combinatorial treatments have been evaluated in various clinical trials. The outcome demonstrated that these therapies increased overall survival in prostate cancer patients. However, the lifespan increment of prostate cancer patients is still challenging for clinicians as all these drug therapies become resistant after a certain time of treatment.

## DECLARATIONS

### Copyright

© The Author(s) 2022.
